# Candidate Genetic Modifiers in Alport Syndrome: A Case Series

**DOI:** 10.3390/life15020298

**Published:** 2025-02-14

**Authors:** Ștefan Nicolaie Lujinschi, Bogdan Marian Sorohan, Bogdan Obrișcă, Alexandra Vrabie, Elena Rusu, Diana Zilișteanu, Camelia Achim, Andreea Gabriella Andronesi, Gener Ismail

**Affiliations:** 1 Department 3, Nephrology, Faculty of Medicine, Carol Davila University of Medicine and Pharmacy, 050474 Bucharest, Romaniaalexandra.vornicu@drd.umfcd.ro (A.V.);; 2 Fundeni Clinical Institute, 022328 Bucharest, Romania

**Keywords:** type IV collagen, Alport syndrome, COL4-related disorders, gene modifiers, podocytes, extracellular matrix

## Abstract

Background: Alport syndrome (AS) is one of the most common monogenic kidney disorders. Recent studies have highlighted the modifier effect of variants involving podocyte and non-collagenous extracellular matrix (ECM) proteins in AS. Methods: We report a case series of eight patients with genetically proven AS and simultaneous variants involving podocyte and non-collagenous ECM proteins. Our aim is to describe the influence of such variants on the phenotype of patients with AS. Results: We identified 10 different type IV collagen variants. Patients were diagnosed with autosomal dominant (3/8), autosomal recessive (2/8), digenic (2/8) and X-linked AS (1/8). There were eight different variants involving podocyte and non-collagenous ECM proteins. The genes involved were CRB2, LAMA5, LAMB2, NUP107, MYO1E and PLCE1. Four patients (LAMB2, LAMA5 and PLCE1 variants) presented with nephrotic syndrome or nephrotic range proteinuria. Two patients had hearing loss. Most patients (7/8) had a family history of kidney disease. Two patients (LAMB2 and LAMA5 variants) were diagnosed with focal segmental glomerulosclerosis. Two patients developed end-stage kidney disease (LAMA5, MYO1E and NUP107 variants). Conclusions: Although mutations of podocyte and ECM proteins do not have phenotypic expression in monoallelic form, the presence of such variants could explain the phenotypic variability of AS.

## 1. Introduction

Alport syndrome (AS), one of the most common monogenic kidney disorders, is caused by variants involving type IV collagen (COL4) genes leading to ultrastructural abnormalities of the glomerular basement membrane (GBM). The classical clinical picture consists of hematuria and progressive kidney dysfunction, eventually leading to end-stage kidney disease (ESKD), sensorineural hearing loss and ocular involvement [1]. The renal phenotype usually follows a series of sequential stages: isolated microscopic hematuria, microalbuminuria, macroalbuminuria and, finally, progressive kidney dysfunction [1,2,3]. Extrarenal involvement is rarely clinically evident at onset, and is usually diagnosed in more advanced stages of the disease [1,2,3].

Males with X-linked forms of AS (XLAS; OMIM ID: 301050 [4]) usually display a more severe phenotype with early-onset ESKD and clinically significant extrarenal involvement [1,2,3]. Autosomal recessive forms (ARAS; OMIM IDs: 620536 [5] and 203780 [6]) tend to have similar outcomes to XLAS, with both males and females being equally affected [7,8]. A milder clinical presentation is reported in patients with autosomal dominant forms (ADAS; OMI IDs: 104200 [9] and 141200 [10]) and in females with XLAS [11,12].

The severity of renal involvement and the presence of extrarenal features are determined by the inheritance pattern and the molecular consequences of each variant [13]. Despite these typical characteristics, there is significant heterogeneity in terms of clinical presentation, with previous reports showing a noteworthy intrafamilial variability [11,12,14]. Moreover, although there is a high diagnostic yield for genetic testing when there is a clinical suspicion of AS, there is also a sizable proportion of patients with chronic kidney disease (CKD) of unknown cause that are subsequently diagnosed with AS [15,16].

Especially with ADAS, clinical outcomes are highly unpredictable, with some patients presenting only with persistent isolated microscopic hematuria, but others showing rapid progression to ESKD [17,18]. As both the clinical and the pathological features of AS can be nonspecific, in the absence of genetic testing, a high proportion of cases are mistakenly diagnosed as primary focal segmental glomerulosclerosis (FSGS) based solely on lesions of focal sclerosis shown by kidney biopsy (KB) [19,20]. As a consequence of this diagnostic error, many patients then receive unnecessary immunosuppressive therapies [19,20]. Furthermore, many reports suggest that AS could be the leading cause of adult-onset FSGS [21,22].

Moreover, the difficulties of AS diagnosis based only on clinical and histological criteria are highlighted by the high proportion of patients that were not suspected to have AS prior to genetic testing [15]. As reported by Groopman et al., in a large cohort of patients with CKD, only 38% of patients with genetically proven AS had suggestive clinical features prior to genetic testing [15]. In the same cohort, 16% of patients with AS were previously diagnosed with FSGS [15].

This overlap of clinical and histological features led to a modification of the current nomenclature, with some sources recommending the use of the term ‘COL4-related disorders’, emphasizing the broad spectrum of this disease [18,23,24,25].

Although some genotype–phenotype correlations have been described, with truncating variants having the worse phenotype when compared to missense variants [26,27,28], the reasons for the great phenotypic variability seen in clinical practice remain a matter of debate. In the case of missense variants, some factors such as the position of the substitution and the type of both the substituted and the substituting amino acid were shown to influence relevant clinical outcomes such as the overall renal survival [14,29].

There is a growing body of evidence related to the worsening of the AS phenotype caused by modifier gene mutations or by variants involving podocyte or extracellular matrix proteins [30,31,32]. It has been proposed that such heterozygous variants, although lacking phenotypic expression in monoallelic form, could have a gene modifier effect or could manifest a digenic inheritance in conjunction with a heterozygous COL4 variant [31]. The alteration of laminin α5 (*LAMA5* genes), podocin (*NPHS2* gene) and non-muscle myosin (*MYO1E* genes) expression has been suggested to have a modifier effect in case series and small cohorts of patients with AS [31,32]. Many other genes were proposed [31], but the clinical relevance of such variants is still unknown.

Therefore, since classical factors such as inheritance and the type of variant are not able to fully predict the outcomes in AS, the effect of gene modifiers remains a possible explanation for the large phenotypic spectrum of AS.

Herein, we report a case series aiming to describe the clinical, pathological and genetic characteristics of patients with COL4-related disorders and concomitant variants involving podocyte and non-collagenous extracellular matrix proteins.

## 2. Materials and Methods

### 2.1. Ethics Approval

The study was approved by the Ethics Committee of Fundeni Clinical Institute (approval number: 66034, date: 15 December 2023). All subjects provided written consent for participation. After reading the manuscript, all the patients granted their approval for publication. All the research was conducted according to the guidelines of the Declaration of Helsinki.

### 2.2. Study Design

We report a case series of 8 patients with genetically proven COL4-related disorders and simultaneous variants involving podocyte or non-collagenous basement membrane proteins. Our main aim was to describe these concomitant non-collagenous variants and to assess their possible influence on the phenotypic spectrum of patients with AS.

We included patients that were over 18 years old and had a positive genetic test for at least one COL4 variant and at least one simultaneous variant involving podocyte or non-collagenous basement membrane proteins (see Section 2.4 Genetic testing).

### 2.3. Clinical Data

Data were collected using the available medical records. The following clinical data were collected: sex, age (at the time of disease onset, at the time of the kidney biopsy and at the time of genetic testing), clinical picture at onset (type of renal syndrome, presence of extrarenal features), family history of kidney disease (including the clinical spectrum and the number of relatives affected), treatment history (use of immunosuppression prior to genetic testing, use of nephroprotective measures) and clinical outcomes (ESKD and the need for kidney replacement therapy). The following laboratory data were collected: serum creatinine (with glomerular filtration rate being estimated using the 2021 CKD Epidemiology Collaboration [CKD-EPI] creatinine equation [33]), 24 h proteinuria and the presence of hematuria.

In cases where a kidney biopsy (KB) was performed, histological data were recorded. Kidney biopsy specimens were examined by light microscopy (LM), immunofluorescence (IF) and electron microscopy (EM).

### 2.4. Genetic Testing

Data were retrospectively collected from the genetic test reports provided by each patient. All patients granted their consent for the reproduction of the genetic test result. Recorded data included gene, type and position of the mutation, inheritance, zygosity and pathogenicity.

Genetic testing was performed by two different accredited laboratories (Blueprint Genetics, Keilaranta 16 A-B, 02150 Espoo, Finland, and Invitae, 1400 16th Street, San Francisco, CA 94103, USA). In all cases, blood samples were collected for genetic testing. Each laboratory used its own validated protocol for DNA extraction, quantity and quality assessment and DNA sequencing. The following gene panels were used: FLEX Nephrotic Syndrome Panel Plus, provided by Blueprint Genetics, and the Invitae Alport Syndrome Panel and Invitae Expanded Renal Disease Panel, both provided by Invitae. In some cases, specific genes were added to the panel by the requesting physician to increase the diagnostic yield.

Variant classification was performed according to the guidelines of the American College of Medical Genetics and Genomics (ACMG) [34], as reported in the genetic test result. The pathogenicity of each variant was assessed by each laboratory. VarSome tools [35] and the ClinVar database [36] were used to further characterize the variants. The following in silico prediction tools were used: AphaMissense, MutaTaster and SIFT. For the interpretation of in silico predictors, the calibrated thresholds available on VarSome [35] were used. For detailing the ACMG criteria, the InterVar database [37] was used.

Regarding the genetic diagnosis of AS, we included patients in whom pathogenic (PV) and likely pathogenic variants (LPV) were considered causative. Patients with variants of uncertain significance (VUSs) were included only if they fulfilled 2 out the following 3 supplementary criteria: (1) clinical picture suggestive of AS, (2) diagnosis of AS, thin basement membrane disease (TBMD) or FSGS on kidney biopsy and (3) pedigree features suggestive of AS. Variants involving podocyte and non-collagenous basement membrane proteins were included irrespective of pathogenicity, zygosity or inheritance.

For variants inherited in both a recessive and dominant manner, inheritance was established based on pedigree data and on previous genetic testing in the patient’s family (if available). Using the current guidelines, the type of AS was assessed based on zygosity, inheritance and gender [18]. Those with multiple COL4 variants involving different genes were classified as having digenic or complex inheritance patterns.

Patients heterozygous for more than one variant involving the same COL4 gene were classified as being either heterozygous or compound heterozygous depending on the position of the mutations, the pedigree and the clinical data. In cases where the laboratory could not determine if two variants involving the same COL4 gene were on the same parental chromosome (in cis) or on different parental chromosomes (in trans), the type of AS was established based on clinical and pedigree data. Particularly, the clinical picture in affected relatives and the rate of progression to ESKD were considered.

Based on previous experience from XLAS, variants were classified in 6 categories depending on the type of mutation: (1) missense, (2) nonsense, (3) frameshift, (4) in-frame deletions or insertions, (5) noncoding and (6) splice junction loss [38,39].

## 3. Results

### 3.1. Clinical Onset

Between January 2020 and August 2024, we identified 68 adults with positive genetic tests for COL4 variants in our database. Out of those, there were eight patients that had concomitant variants affecting the integrity of podocytes and/or the filtration barrier. Five of them (5/8) were females. The age at onset ranged between 23 and 42 years old. The general clinical and pedigree characteristics of our patients are detailed in Table 1.

The most frequent renal syndrome at onset was nephritic syndrome (4/8), followed by nephritic–nephrotic syndrome (2/8), nephrotic syndrome (1/8) and nephrotic range proteinuria (1/8). Estimated glomerular filtration rate (eGFR) ranged between 70 and 111 mL/min/1.73 m^2^ at onset, while proteinuria ranged between 0.8 and 22 g/day. Only one patient did not present hematuria at onset. There was only one patient with episodes of macroscopic hematuria, and they were mistakenly diagnosed as having IgA nephropathy (see detailed description of the cases in Section 4.). Only two patients presented with hearing loss. Another two subjects had multiple bilateral renal cysts.

Most of the patients had a positive family history for kidney disease (7/8). Their relatives presented with CKD of unknown cause (3/7), nephritic syndrome (2/7), nephrotic syndrome (1/7) or isolated hematuria (1/7). Only three cases had relatives that were also diagnosed with AS based on kidney histology and/or genetic testing.

### 3.2. Pathological Characteristics

There were five patients (5/8) that underwent a KB. Pathological findings are summarized in Table 1. Our patients’ ages at the time of KB ranged from 23 to 44 years old. Based on KB, our patients were diagnosed with either TBMD (3/5; N3, N5 and N7) or FSGS (2/5; N1 and N4). IF was only available for two patients. One of those had positive staining for C3 in the mesangium, while the other had negative IF. In all cases where EM was available (4/5), structural defects of the GBM were observed: either thinning (3/4; the patients diagnosed with TBMD) or thickening of the GBM (1/4). Three subjects (3/4) displayed focal effacement of podocyte foot processes.

### 3.3. Genetic Characteristics

There were 10 different COL4 variants identified. Half of them (5/10) were VUSs and the other half comprised one risk variant, two PV and two LPV. Four patients had multiple COL4 variants: two related patients had digenic AS (N1 and N2; digenic *COL4A3*/*A4*) and two unrelated subjects had ARAS (compound heterozygous). Regarding the classification of AS, there were three cases of ADAS (N4, N5 and N6), two cases of ARAS (compound heterozygous; N7 and N8), two cases of digenic AS (N1 and N2) and one case of XLAS (in a female; N3). In both cases of ARAS, the laboratory could not determine if the two variants were in cis or in trans, so the classification was performed considering clinical and pedigree data. There were five missense variants, three in-frame (two deletions and one insertion) variants, one noncoding variant and one splice site junction loss variant. We identified four variants that, to our knowledge, were not previously reported in the literature.

We identified eight different variants involving podocyte and/or non-collagenous basement membrane proteins. There was one patient with two such different variants (N8). The following genes were involved: *LAMB2* (two variants; N1, N2 and N5), PLCE1 (two variants; N6 and N7), *CRB2* (one variant; N3), *LAMA5* (one variant; N4), *NUP107* (one variant; N8) and MYO1E (one variant; N8). All the variants were heterozygous and were inherited in an autosomal recessive manner. There were only VUSs detected. All variants identified were missense mutations. To our knowledge, only one variant was previously described.

**Figure 1:**
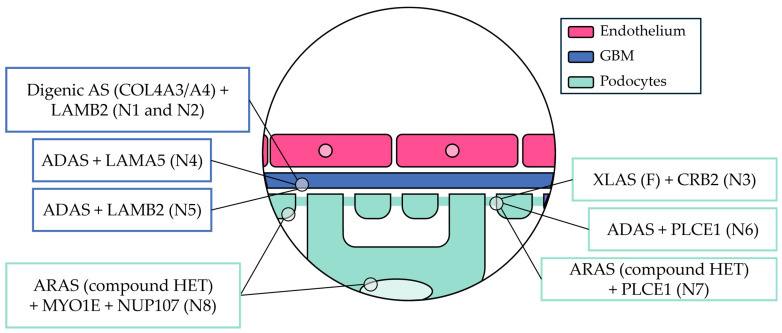
The compartment of the glomerular filtration barrier affected in each case. ADAS—autosomal dominant Alport syndrome, ARAS—autosomal recessive Alport syndrome, AS—Alport syndrome, COL4—type IV collagen, *COL4A3*—α3 chain of type IV collagen, *COL4A3*—α4 chain of type IV collagen, *CRB2*—crumbs cell polarity complex component 2, F—female, GBM—glomerular basement membrane, HET—heterozygous, *LAMA5*—laminin subunit α5, *LAMB2*—laminin subunit β2, *MYO1E*—myosin 1E, N—number, *NUP107*—nucleoporin 107, *PLCE1*—phospholipase Cε1, XLAS—X-linked Alport syndrome.

The above-mentioned variants involved slit diaphragm proteins (three variants; N3, N6 and N7), non-collagenous GBM proteins (three variants; N1, N2, N4 and N5), podocyte nuclear proteins (one variant; N8) and podocyte cytoskeletal proteins (one variant; N8). The glomerular filtration barrier compartment involved in each case is depicted in Figure 1. The detailed characteristics of each variant are depicted in Table S1. The references for the variants that have been previously described in the literature are presented in Table S1 [7,15,28,40,41,42,43]. The detailed in silico analysis of the identified variants is presented in Table S2.

### 3.4. Treatment Record

Therapeutic strategies used in our patients are detailed in Table S3.

### 3.5. Follow-Up

The duration of follow-up, between clinical onset and the latest evaluation, ranged from 6 months to 17 years. During this period, two patients developed ESKD (N4 and N8). One received a living-donor kidney transplant (LDKT) at 29 years old (N4) and the other started hemodialysis at 39 years old (N8) and is currently on the transplant waiting list. During follow-up, most of the patients who did not reach ESKD (5/6) experienced a decline in eGFR ranging from −1.29 to −6.75 mL/min/1.73 m^2^ per year (mean −3.77 ± 2.28 mL/min/1.73 m^2^ per year). One case displayed a rise in eGFR (+7 mL/min/1.73 m^2^; N2), but it is worth mentioning that the follow-up was short (6 months). There was a decrease in proteinuria ranging from −0.5 to −1.9 g/day (N2, N3, N5 and N7). Patient N1 was the only one who experienced an increase in proteinuria (+0.83 g/day). Data regarding proteinuria were not available for one patient (N6) and were deemed not relevant to those who reached ESKD (N4 and N8). The clinical outcomes in each case are represented in Table S4.

## 4. Discussion

### 4.1. Non-Collagenous Extracellular Matrix Proteins

#### 4.1.1. Laminin Subunit β2—LAMB2

Alongside type IV collagen, the GBM contains other extracellular matrix proteins such as laminin. The specific isoform included in the structure of the GBM is laminin 521, composed of α5, β2 and γ1 chains. The *LAMB2* gene encodes the β2 chain [44]. Homozygous or compound heterozygous variants in the *LAMB2* gene are associated with Pierson syndrome, defined by congenital nephrotic syndrome and ocular and neuromuscular abnormalities [44]. The complete clinical picture is characteristic of variants with significant molecular impact (i.e., truncating mutations), while missense variants can exhibit an incomplete and less severe clinical picture [45]. There is no phenotype associated with heterozygous variants, as these patients are considered carriers [45].

Murine studies have highlighted *LAMB2* as a possible modifier gene in AS [46]. It has been well documented that monoallelic *LAMB2* variants can increase the rate of kidney disease progression in murine models of AS [46]. Funk et al. showed that, in murine models of both ARAS and XLAS, only one mutant allele of *LAMB2* can worsen both the clinical outcomes and the histological lesions [46]. It has been hypothesized that the alteration of laminin network polymerization in conjunction with the defective COL4 structure leads to the destabilization of the GBM, accelerating the rate of progression [46]. Although the experimental research is promising, there are no cases of heterozygous *LAMB2* variants influencing the AS phenotype described in the literature.

In our cohort, we identified three patients with AS and concomitant heterozygous *LAMB2* variants (N1, N2 and N5; see Table S1). Patient N1 presented with nephritic syndrome and was diagnosed with primary FSGS based on KB. She received immunosuppression with no clinical response. As proteinuria increased, she was classified as having steroid-resistant FSGS and was referred for genetic testing. One *COL4A3* risk variant and one *COL4A4* VUS were identified. Although there were no PV or LPV identified, considering the clinical and the histological features, she was classified as having digenic AS.

Patient N2 was screened as she was the sister of patient N1. She was diagnosed with nephritic syndrome. Subsequent genetic testing showed the same variants as her sibling. There are limited data to assess the clinical evolution (only 6 months of follow-up).

Case N5 presented with nephrotic range proteinuria. KB was performed, and she was subsequently diagnosed with TBMD. There were no sclerotic glomeruli in LM, nor podocyte abnormalities in EM. She exhibited some atypical features such as episodes of macroscopic hematuria in early adulthood and the presence of multiple bilateral kidney cysts. Although both characteristics have been described in AS, the episodes of macroscopic hematuria tend to appear mostly during childhood [47] and kidney cysts tend to more often affect older patients with advanced CKD [48].

Both cases that had extensive follow-up (N1 and N5) showed the persistence of proteinuria and the progressive deterioration of renal function despite nephroprotective care.

#### 4.1.2. Laminin Subunit α5—LAMA5

Increased expression and synthesis of both laminin α1 and α5 have been previously described in murine models of AS [49]. Abrahamson et al. showed that the disruption of the normal laminin network contributes to the abnormal permeability of the GMB in *COL4A3* knock-out mice [49]. This finding highlights the role of laminin in the maladaptive response caused by the defective COL4 network.

In accordance with the organ tropism of laminin α5, the clinical presentation of *LAMA5*-related disorders can include severe developmental abnormalities affecting the kidney, eyes, lungs and limbs [50,51]. Isolated renal involvement, presenting with autosomal recessive nephrotic syndrome, FSGS or cystic phenotype, has also been described [52,53]. Although there are differences in disease severity depending on the molecular impact of the mutation, phenotype–genotype correlations are yet to be described [51,52,53]. It is unclear if monoallelic *LAMA5* variants can be clinically significant, as severe forms of disease have also been reported in heterozygous patients [54,55].

There was only one patient with concomitant *LAMA5* mutations (N4) in our cohort. He presented with nephritic–nephrotic syndrome at the age of 24. Based on KB, he was diagnosed with primary FSGS. EM showed concomitant ultrastructural defects of the GBM, and focal podocyte foot process effacement, suggesting a secondary form of FSGS. He received immunosuppression with no clinical response. He reached ESKD 5 years later and started hemodialysis (HD). Genetic testing was requested as part of the assessment required for kidney transplantation. Although only a VUS involving the *COL4A3* gene was detected, considering the KB result and pedigree data, he was diagnosed with ADAS (see Table S1). He later received an LDKT from his maternal aunt.

The clinical evolution was deemed atypical as the rate of progression and the degree of proteinuria are not characteristic of ADAS. We hypothesized that the *LAMA5* variant could have influenced the clinical picture. As no other relative underwent genetic testing, it is not possible to distinguish digenic inheritance from a gene modifier effect.

### 4.2. Slit Diaphragm Proteins

#### 4.2.1. Crumbs Cell Polarity Complex Component 2—CRB2

Encoded by *CRB2* gene, Crumbs2 is an important regulator of both cellular polarity and intercellular contacts [56]. Murine studies have shown that Crumbs2 organizes in clusters adjacent to Nephrin at the site of the slit diaphragm, highlighting the role of Crumbs2 in maintaining the normal architecture of the slit diaphragm [57]. In fact, loss of function variants of *CRB2* can lead to steroid-resistant nephrotic syndrome resembling the clinical spectrum of Nephrin mutations [58]. Other clinical features include cerebral ventriculomegaly, a rise in serum α-fetoprotein, cystic kidney disease and, rarely, cardiac malformations [58]. To date, there are no reports of monoallelic *CRB2* variants influencing the clinical outcomes in AS.

Patient N3 was a female with XLAS that displayed simultaneous monoallelic *CRB2* mutation. At onset, she presented with nephritic syndrome. KB supported the diagnosis of TBMD. EM showed focal podocyte injury, although proteinuria was under 1 g/day. During follow-up, she maintained normal renal function and experienced a significant drop in proteinuria after maximal renin–angiotensin system blockade.

#### 4.2.2. Phospholipase C ε1—PLCE1

Although not included in the structure of the slit diaphragm, phospholipase C ε1 (encoded by *PLCE1*) is an important enzyme involved in linking the activity of slit diaphragm proteins with the podocyte cytoskeleton [59,60]. Through intracellular signaling, phospholipase C ε1 is also involved in the migration, proliferation and differentiation of podocytes [59].

The severity spectrum of *PLCE1*-realted disease is broad, with some homozygous or compound heterozygous patients presenting with diffuse mesangial sclerosis, some with genetic FSGS and a minority of cases being asymptomatic [61,62]. There is no case of monoallelic *PLCE1* mutation influencing the phenotype of AS reported in the literature.

There were two patients (N6 and N7) with monoallelic *PLCE1* variants in our cohort. One was classified as having ADAS and the other one as having ARAS (compound heterozygous; see Table S1). Both patients had nephrotic proteinuria at onset. In the case of patient N6, the presence of kidney dysfunction and the lack of hematuria could be consistent with the natural evolution of AS. However, proteinuria rarely reaches the nephrotic range in ADAS [14]. Unlike N6, patient N7 was diagnosed with nephritic–nephrotic syndrome in early adulthood and had normal renal function, in contrast with the typical sequential evolution of AS. Only patient N7 underwent a KB. Despite the absence of glomerulosclerosis, there were signs of podocyte injury in EM. Both patients progressed in terms of kidney function deterioration, losing 31% and 61% of baseline eGFR over a follow-up of 9 and 15 years, respectively (eGFR loss of −1.66 and −4.53 mL/min/1.73 m^2^ per year, respectively). These data suggest a modifier effect rather than a digenic inheritance, but extensive research is necessary to confirm this hypothesis.

### 4.3. Podocyte Cytoskeletal Proteins

Myosin 1E—MYO1E

*MYO1E* encodes myosin 1E, a podocyte cytoskeletal protein belonging to non-muscle class I myosin. As myosin 1E participates in maintaining podocyte foot processes’ normal architecture [63,64], mutation in *MYO1E* can cause autosomal recessive nephrotic syndrome [65]. The physiological role of *MYO1E* is not limited to podocyte cytoskeletal integrity as it has been shown that Myo1E knock-out mice exhibit ultrastructural defects of the GBM similar to AS, suggesting a role in regulating the podocyte–extracellular matrix crosstalk [66].

Studying a consanguineous Pakistani family, Lennon et al. showed that biallelic *MYO1E* variants can alter the phenotype of XLAS [67]. Those who had concomitant *COL4A5* and *MYO1E* mutations presented with nephrotic syndrome and rapidly progressed to ESKD, in contrast to those that only harbored the *COL4A5* variant [67]. The phenotype was severe even in a female with XLAS. Those that had only one mutant allele of *MYO1E* associated with XLAS had a milder clinical picture [67]. The authors concluded that the phenotype was a consequence of the two distinct genetic disorders, rather than one mutation enhancing the other, pleading for a digenic inheritance of *COL4A5* and *MYO1E* variants in this case [67].

Case N8, who was diagnosed with ARAS (compound heterozygous) and had concomitant mutations in both *MYO1E* and *NUP107*, is discussed in the following section.

### 4.4. Podocyte Nuclear Proteins

Nucleoporin 107—NUP107

Nucleoporins are important components of the nuclear pore complex (NPC), a central structure in nucleocytoplasmic transport [68]. Biallelic variants in *NUP107*, encoding nucleoporin 107, one of the structural subunits of the NPC, are known to cause childhood-onset nephrotic syndrome [68]. The molecular mechanism by which the dysfunction of nuclear envelope proteins leads to nephrotic syndrome is not fully understood. It has been hypothesized that it involves an unknown link between nuclear proteins and the actin cytoskeleton [68,69]. Nevertheless, the clinical spectrum in homozygous *NUP107* mutation is severe, with patients reaching ESKD in early childhood and some having diverse developmental disorders [68,69]. No link between *NUP107*-related disorders and AS has been described.

In our cohort, there was one patient (N8) that had both monoallelic *MYO1E* and *NUP107* variants. He was classified as having ARAS (compound heterozygous). Little is known about the clinical onset, as he had already reached ESKD when he presented to our clinic. He had no significant prior medical history. Ten years before reaching ESKD and starting HD, he had an eGFR of 70 mL/min/1.73 m^2^ and displayed nephritic syndrome. His sister also reached ESKD in early adulthood. In her case, genetic testing showed only one variant involving the *COL4A3* gene (c.1814G>T). The genetic testing panel did not include *MYO1E* or *NUP107*. She later underwent a deceased-donor kidney transplantation (DDKT). Genetic testing was requested for patient N8 as part of the kidney transplantation assessment. He is currently on the waiting list for a DDKT.

Considering that the clinical course is consistent with ARAS and that the patient’s sister had a similar evolution, although she had only one mutant allele in *COL4A3* (with *MYO1E* and *NUP107* not tested), it is not possible to determine whether there was any modifier effect.

## 5. Conclusions

We conducted a case series study analyzing eight patients with AS and concomitant mutations involving podocyte or non-collagenous extracellular matrix proteins. Some of these variants were already identified as candidate genetic modifiers in human (*LAMA5* and *MYO1E*) or in murine models of AS (*LAMB2*), and some—although not described in the literature—could mechanistically influence the clinical and histological spectrum in AS (*PLCE1*, *CRB2* and *NUP107*).

As our study was based on medical records and genetic pedigree was usually not available, it is inconclusive if the variants detected possess a modifier effect. Nevertheless, as some patients presented an atypical clinical course, our results reinforce the idea that the phenotypic spectrum of AS could be influenced by concomitant mutant alleles involving podocyte or non-collagenous extracellular matrix proteins. Future studies should thoroughly assess the extent of this effect.

## Figures and Tables

**Table 1:** Clinical and histological characteristics of patients included in our study.

N.		1	2	3	4	5	6	7	8
Gender		Female	Female	Female	Male	Female	Female	Male	Male
**Clinical Picture**	**Age—Onset (y.)**	23	29	29	24	36	42	23	28
	**Renal Syndrome**	Nephritic syndrome	Nephritic syndrome	Nephritic syndrome	Nephritic-nephrotic syndrome	Nephrotic range proteinuria	Nephrotic syndrome	Nephritic-nephrotic syndrome	Nephritic syndrome
	**eGFR—Onset (mL/min/1.73m^2^)**	111	101	101	72	75	48	111	70
	**Proteinuria—Onset (g/day)**	0.95	3	0.8	22.0	4.6	3.5	3.6	NA
	**Hematuria—Onset**	Microscopic	Microscopic	Microscopic	Microscopic	Episodes of macroscopic hematuria	Absent	Microscopic	Microscopic
	**Extrarenal Features**	Absent	Absent	Absent	Absent	Hearing loss	Absent	Absent	Hearing loss
	**Kidney Cysts**	No	No	No	No	Yes	Yes	No	No
**Family History**	**Positive Family History**	Yes	Yes	Yes	Yes	Yes	No	Yes	Yes
	**Clinical Picture**	Nephritic syndrome	Nephritic syndrome	CKD	Nephrotic syndrome	CKD/KRT	-	Isolated microscopic hematuria	KRT
	**Genetic/** **Histologic** **Diagnosis of AS**	Yes	Yes	No	No	No	-	No	Yes
**Kidney Biopsy**	**Kidney Biopsy**	Yes	No	Yes	Yes	Yes	No	Yes	No
	**Age—Kidney Biopsy (y.)**	23	-	31	24	44	-	32	-
	**Diagnosis**	FSGS	-	TBMD	FSGS	TBMD	-	TBMD	-
	**GBM Alterations**	No EM available	-	Thin GBM	Thick GBM	Thin GBM	-	Thin GBM	-
	**GS**	Yes (focal)	-	No	Yes (focal)	No	-	No	-
	**IF**	NA	-	NA	NA	Negative	-	Positive (+) staining for C3 (mesangial)	-
	**Podocyte Foot** **Effacement**	No EM available	-	Focal	Focal	Absent	-	Focal	-

AS—Alport syndrome, CKD—chronic kidney disease, eGFR—estimated glomerular filtration rate, EM—electron microscopy, FSGS—focal segmental glomerulosclerosis, GBM—glomerular basement membrane, GS—glomerulosclerosis, IF—immunofluorescence, KRT—kidney replacement therapy, N.—number, NA—not available, y.—year, TBMD—thin basement membrane disease.

## Data Availability

The datasets used during the current study are available from the corresponding author on reasonable request.

## Supplementary Materials

The following supporting information can be downloaded at https://www.mdpi.com/article/10.3390/life15020298/s1: Table S1: Genetic testing results; Table S2: In-Silico analysis of proposed candidate variants involving podocyte and/or non-collagenous basement membrane proteins; Table S3: Treatment history for each patient. Table S4. Clinical evolution during the available follow-up.
