# Supplementary material for: Candidate Genetic Modifiers in Alport Syndrome: A Case Series
Source: Life (Basel). 2025 Feb 14;15(2):298. doi: 10.3390/life15020298 (PMC11857524; doi:10.3390/life15020298)
Supplement: Supplementary file 1 [file life-15-00298-s001.zip › life-3339776-supplementary.pdf]

Article

Candidate Genetic Modifiers in Alport Syndrome: A Case Series

1. Supplementary Materials

1.1. Supplementary Table S1

Table S1. Genetic testing results.

Number	Genetic test	Reasons for testing	Age—genetic testing (y.)	COL4 Gene										Proposed Modifier Gene									
				**Gene**	**Position**	**Nucleotide change**	**Impact**	**Zygosity**	**Inheritance**	**Consequence**	**Pathogenicity**	**Type of AS**	**Previously described**	**Gene**	**Position**	**Nucleotide change**	**Amino acid change**	**Zygosity**	**Inheritance**	**Consequence**	**Pathogenicity**	**Compartment**	**Previously described**
1	Nephrotic syndrome panel	Steroid resistant FSGS	25	*COL4A4*	Exon 28	c.2171G>A	p.Arg724His	HET	AR, AD	Missense	VUS	Digenic	No *	*LAMB2*	Exon 9	c.1114G>C	p.Gly372Arg	HET	AR	VUS	Missense	GBM	No *
				COL4A3	Exon 28	c.2083G>A	p.Gly695Arg	HET	AR, AD	Missense	Risk factor		Yes [40]										
2	Family variant testing	Family history	28	*COL4A4*	Exon 28	c.2171G>A	p.Arg724His	HET	AR, AD	Missense	VUS	Digenic	No *	*LAMB2*	Exon 9	c.1114G>C	p.Gly372Arg)	HET	AR	VUS	Missense	GBM	No *

4<br>Extended renal disease panel<br>Structural effect of GBM, nephrotic–nephritic sd.<br>29	3<br>Nephrotic syndrome panel<br>Structural defect of GBM<br>31	
COL4A3<br>Exon 41<br>c.3546_3548dup<br>p.Gly1183dup<br>HET<br>AR, AD<br>In-frame insertion<br>VUS<br>ADAS<br>Yes [15]	*COL4A5*<br>Deletion of exon 2<br>c.(81+1_82-1)_(141+1_142-1)del (141+1_142-1)del<br>Partial gene<br>HET<br>X-linked<br>In-frame deletion<br>LPV<br>XLAS (female)<br>Yes [28]	*COL4A3*<br>Exon 28<br>c.2083G>A<br>p.Gly695Arg<br>HET<br>AR, AD<br>Missense<br>Risk factor<br><br>Yes [40]
LAMA5<br>Exon 75<br>c.10359C>G<br>p.His3453Gln<br>HET<br>AR<br>VUS<br>Missense<br>GBM<br>No *	*CRB2*<br>Exon 13<br>c.3665T>C<br>p.Leu1222Pro<br>HET<br>AR<br>VUS<br>Missense<br>Podocyte—slit diaphragm<br>No *	

5	Extended renal disease panel	Structural defect of GBM, proteinuria	52	*COL4A4*	Exon 20	c.1321_1369+3del	Partial deletion of exon 20	HET	AR, AD	Splice site	PV	ADAS	Yes [41]	*LAMB2*	Exon 12	c.1540C>T	p.His514Tyr	HET	AR	VUS	Missense	GBM	No *
6	Nephrotic syndrome panel	Nephrotic sd.	50	*COL4A4*	Exon 48	c.5045G>A	p.Arg1682Gln	HET	AR, AD	Missense	VUS	ADAS	Yes [42]	*PLCE1*	Exon 3	c.1223G>T	p.Arg408Ile	HET	AR	VUS	Missense	Podocyte—slit diaphragm	Yes [43]
7	Extended renal disease panel	Structural defect of GBM	36	*COL4A4*	Intron 32	c.2969-10A>G	Intron 32	HET	AR, AD	Noncoding	VUS	ARAS—compound heterozy-	No *	*PLCE1*	Exon 31	c.6696C>G	p.Ile2232Met	HET	AR	VUS	Missense	Podocyte—slit diaphragm	No *

				COL4A4	Exon 40	c.3781G>A	p.Gly1261Arg	HET	AR, AD	Missense	VUS		No *
8	Nephrotic syndrome panel	ESKD, hearing loss, family history of CKD	48	*COL4A3*	Exon 26	c.1814G>T	Gly605Val	HET	AR, AD	Missense	LPV	ARAS—compound heterozygous **	No *
				COL4A3	Exon 1	c.40_63del	p.Leu14_Leu21del	HET	AR, AD	In-frame deletion	PV		Yes [7]
				MYO1E	Exon 2	c.85A>G	p.Lys29Glu	HET	AR	VUS	Missense	Podocyte—cyto-skeleton	No *
				NUP107	Exon 3	c.161G>A	p.Arg54Gln	HET	AR	VUS	Missense	Podocyte—nuclear protein	No *

AD—autosomal dominant, ADAS—autosomal dominant Alport syndrome, AR—autosomal recessive, ARAS—autosomal recessive Alport syndrome, AS—Alport syndrome, CKD—chronic kidney disease, COL4—type IV collagen, *CRB2*—crumbs cell polarity complex component 2, ESKD—end-stage kidney disease, FSGS—focal segmental glomerulosclerosis, GBM—glomerular basement membrane, HET—heterozygous, *LAMA5*—laminin subunit α5, *LAMB2*—laminin subunit β2, LPV—likely pathogenic variant, *MYO1E*—myosin 1E, *NUP107*—nucleoporin 107, *PLCE1*—phospholipase C ε1, PV—pathogenic variant, sd.—syndrome, VUS—variant of uncertain significance, XLAS—X-linked Alport syndrome, y.—years. * VarSome tools [35] and the ClinVar database [36] were used to assess each variant. ** In cases where the laboratory could not determine if two variants involving the same COL4 gene were on the same parental chromosome (in cis) or on different parental chromosomes (in trans), the type of Alport syndrome was established based on clinical and pedigree data.

1.2. Supplementary Table S1

Table S2. In-Silico analysis of proposed candidate variants involving podocyte and/or non-collagenous basement membrane proteins

Gene	Nucleotide change	Molecular impact	gnom AD exomes allele frequency	In-Silico prediction tools*	LOVD—clinical classification	ClinVar significance	ACMG criteria**
CRB2	c.3665T>C	p.Leu1222Pro	0.000306	AlphaMissense: Benign Supporting<br>MutationTaster: Uncertain<br>SIFT: Uncertain	Not reported	Uncertain significance	Uncertain significance (PM2, PP3)
LAMA5	c.10359C>G	p.His3453Gln	0.000269	AlphaMissense: Benign Strong<br>MutationTaster: Uncertain<br>SIFT: Benign Moderate	Not reported	Uncertain significance	Uncertain significance (PP2, BP4)
LAMB2	c.1114G>C	p.Gly372Arg	Not reported	AlphaMissense: Uncertain<br>MutationTaster: Uncertain<br>SIFT: Pathogenic Supporting	Not reported	Not reported	Uncertain significance (PM1, PM2, PP3)
LAMB2	c.1540C>T	p.His514Tyr	Not reported	AlphaMissense: Benign Moderate<br>MutationTaster: Uncertain<br>SIFT: Benign Moderate	Not reported	Uncertain significance	Uncertain significance (PM1, PM2)
MYO1E	c.85A>G	p.Lys29Glu	Not reported	AlphaMissense: Uncertain<br>MutationTaster: Uncertain<br>SIFT: Uncertain	Not reported	Not reported	Uncertain significance (PM1, PM2, PP3)
NUP107	c.161G>A	p.Arg54Gln	0.000493	AlphaMissense: Benign Moderate<br>MutationTaster: Uncertain<br>SIFT: Benign Moderate	Uncertain significance	Uncertain significance	Uncertain significance (PM2)
PLCE1	c.1223G>T	p.Arg408Ile	0.0000201	AlphaMissense: Benign Moderate<br>MutationTaster: Benign Supporting<br>SIFT: Pathogenic Supporting	Not reported	Uncertain significance	Uncertain significance (PM1, PM2)
PLCE1	c.6696C>G	p.Ile2232Met	0.00000401	AlphaMissense: Uncertain<br>MutationTaster: Uncertain<br>SIFT: Pathogenic Supporting	Not reported	Uncertain significance	Uncertain significance (PM1, PM2, BP4)

ACGM—American College of Medical Genetics and Genomics, *CRB2*—crumbs cell polarity complex component 2, gnomAD—Genome Aggregation Database, *LAMA5*—laminin subunit α5, *LAMB2*—laminin subunit β2, LOVD—Leiden Open (source) Variation Database, *MYO1E*—myosin 1E, *NUP107*—nucleoporin 107, *PLCE1*—phospholipase C ε1. * For the interpretation of in-silico predictors, the calibrated thresholds available on VarSome(see reference [35] in the main text)were used. **For detailing the ACMG criteria, the InterVar(see reference [37] in the main text) database was used.

1.3. Supplementary Table S2

Table S3. Treatment history for each patient

TREATMENT					
SGLT2i use	MRB use	Number of nephroprotective drugs (RASi, SGLT2i and/or MRB)	Maximal nephroprotection (at least RASi + SGLT2i)*	Number of antihypertensive drugs (including RASi if hypertensive)	History of immuno-suppression
Yes	No	2	Yes	1	Yes
No	No	0	No	No HTN	No
No	No	1	No	2	No
No	No	1	No	3	Yes
No	Yes	2	No	2	No
Yes	No	2	Yes	No HTN	Yes
Yes	Yes	3	Yes	No HTN	Yes
No	No	0	No	NA	NA

N.	Gender	RASi use	Current RASi dose (% of maximum dose)	Reasons for not reaching maximum RASi dose
1	Female	Yes	50	Orthostatic hypotension
2	Female	No	-	-
3	Female	Yes	100	-
4	Male	Yes	50	Hyperkalemia
5	Female	Yes	100	-
6	Female	Yes	25	Orthostatic hypotension
7	Male	Yes	100	-
8	Male	No	-	-

HTN—hypertension, MRB—mineralocorticoid receptor blocker, N.– number, NA—not available, RASi—renin-angiotensin system inhibitor, SGLT2i—sodium-glucose cotransporter 2 inhibitor, y.—year. *Maximal nephroprotection wasdefined as the association between a RASi—maximum tolerated dose—and SGLT2i use, with or without a MRB.

1.4. Supplementary Table S3

Table S4. Clinical evolution during the available follow-up.

OUTCOME			
Proteinuria decline (g/day)—from onset to the last control	KRT	Age at the start of KRT	Type of KRT
+0.83	No	-	-
-1.58	No	-	-
-0.5	No	-	-
Not relevant – kidney transplant recipient	Yes	29	LDKT – maternal aunt
-1	No	-	-
NA	No	-	-
-1.9	No	-	-
Not relevant—on HD for more than 10 years at the time of the last control	Yes	39	HD

N.	Gender	Extent of follow up—from onset to the last control	eGFR decline (ml/min/1.73m^2 per year)—from onset to the last control
1	Female	4 years	-6.75
2	Female	6 months	+7 ml/min/1.73m^2 in 6 months *
3	Female	3 years	-4.66
4	Male	7 years	Not relevant—kidney transplant recipient
5	Female	17 years	-1.29
6	Female	9 years	-1.66
7	Male	15 years	-4.53
8	Male	11 years	Not relevant—on HD for more than 10 years at the time of the last control

eGFR—estimated glomerular filtration rate, HD—hemodialysis, KRT—kidney replacement therapy, LDKT—living donor kidney transplantation, N.– number. *The length of follow-up was under one year.
